# Human transitional and IgM^low^ mature naïve B cells preserve permissive B‐cell receptors

**DOI:** 10.1111/imcb.12478

**Published:** 2021-06-04

**Authors:** Zhiguo Zhang, Christopher J Jara, Mandeep Singh, Huji Xu, Christopher C Goodnow, Katherine JL Jackson, Joanne H Reed

**Affiliations:** ^1^ Garvan Institute of Medical Research Sydney NSW Australia; ^2^ Department of Rheumatology and Immunology Shanghai Changzheng Hospital Second Military Medical University Shanghai China; ^3^ Faculty of Medicine St. Vincent’s Clinical School UNSW Sydney NSW Australia; ^4^ School of Medical Sciences and Cellular Genomics Futures Institute UNSW Sydney NSW Australia

**Keywords:** antibodies, B‐cell receptor, B cells, immune tolerance, V(D)J recombination

## Abstract

The level of immunoglobulin M (IgM) displayed on the surface of peripheral blood B cells exhibits a broad dynamic range and has been associated with both development and selection. To determine whether IgM surface expression associates with distinct immunoglobulin heavy‐chain (IGH) repertoire properties, we performed deep IgM sequencing of peripheral blood transitional and mature naïve B cells in the upper and lower quartiles of surface IgM expression for 12 healthy donors. Mature naïve B cells within the lowest quartile for surface IgM expression displayed more diverse IGH features including increased complementarity‐determining region 3 length, IGHJ6 segment usage and aromatic amino acids compared with mature naïve B cells with high surface IgM. There were no differences between IGH repertoires for transitional B cells with high or low surface IgM. These findings suggest that a selection checkpoint during progression of transitional to mature naïve B cells reduces the breadth of the IGH repertoire among high surface IgM B cells but that diversity is preserved in B cells expressing low levels of surface IgM.

## Graphical abstract

Self‐tolerance mechanisms tend to remove antibodies with long complementarity‐determining region 3 sequences that are enriched in aromatic amino acids, which has the potential to create holes in the B‐cell repertoire, making us vulnerable to pathogens that mimic self. We reveal a population of circulating mature naïve B cells with low surface immunoglobulin M that preserve these permissive antibody properties in healthy people.

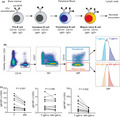

## INTRODUCTION

The rearrangement of immunoglobulin (Ig) variable (V), diversity (D) and junction (J) gene segments in developing B cells is used by all jawed vertebrates to achieve a diverse antibody repertoire capable of recognizing an almost infinite number of pathogens.^1^ For most mammals, molecular recognition of antigens occurs at the third heavy‐chain complementarity‐determining region (HCDR3), composed of hypervariable loops spanning the V–D and D–J junctions. Further diversity is achieved at these gene segment junctions through exonuclease activity and N and P nucleotide addition, making HCDR3 the most diverse region in the antibody repertoire. However, the somewhat random and imprecise mechanisms to achieve diversity can inevitably produce B cells that recognize self‐antigens and multiple tolerance checkpoints exist to prevent self‐reactive B cells from causing autoimmunity.^2^, ^3^, ^4^ Nevertheless, the importance of maintaining antibody repertoire diversity is reflected by the multitude of diversification strategies employed across species to achieve a striking breadth of HCDR3 sequence length and amino acid (AA) composition. For example, chickens and ducks employ gene conversion events with pseudo V segments and longer HCDR3 sequences with higher cysteine and lower tyrosine content to compensate for limitations in V(D)J rearrangements because of only having one functional H chain V and J segment.^5^, ^6^ Similarly, the Australian platypus Ig repertoire has fewer functional V segments compared with other mammals but uses long and highly varied D segments with N nucleotide additions to achieve diversity.^7^ Cattle accomplish antibody diversity by producing an Ig repertoire with the broadest distribution of HCDR3 lengths including short (< 10 residues), long (11–47 residues) and ultralong (> 48 residues).^8^, ^9^ Ultralong HCDR3 sequences are created using a 149‐nucleotide germline D segment (IGHD2) encoding multiple cysteine residues, which maintain a complex structure through the formation of disulfide bonds.^10^

Long HCDR3 sequences (> 24 residues) in the human B‐cell repertoire are primarily generated by recombination events during B‐cell development and have increased usage of the longest germline D and J gene segments (D2–2, D2–15 and J6) with N and P additions.^11^ Among the longest HCDR3 sequences reported in humans are the broadly neutralizing anti‐HIV antibodies. Long HCDR3 loops composed of 20–34 AAs appear advantageous to penetrate the glycan shield of the HIV Env trimer and target gp120.^12^, ^13^, ^14^ Despite beneficial binding to specific microbes, long HCDR3 sequences are relatively rare in humans with only 3.5% of the naïve B‐cell pool containing HCDR3 over 24 residues and 0.43% with HCDR3 over 28 residues.^11^ Moreover, there is an increased frequency of long HCDR3 in out‐of‐frame Ig rearrangements compared with the selected antibody repertoire, which implies that the HCDR3 sequence breadth that can be created may not be available in the mature naïve B‐cell compartment.^15^ The paucity of long HCDR3 in humans may be due to the increased propensity of long HCDR3 sequences to be self‐ or poly‐reactive^16^, ^17^ and raises questions about how HCDR3 breadth is preserved in human antibody repertoires.

A potential mechanism to preserve HCDR3 diversity while mitigating the risk of autoimmunity is to maintain populations of B cells with diverse HCDR3 properties, associated with auto/poly‐reactivity, in a functionally silenced state such as anergy. Anergic B cells bearing autoantibodies are capable of participating in productive immune responses by undergoing somatic hypermutation to remove autoreactivity and increase affinity to the foreign antigen.^18^, ^19^, ^20^ Anergic B cells were first characterized in mice as having low surface IgM with normal levels of surface IgD.^21^ Equivalent populations have been observed in the mature naïve peripheral B‐cell compartment of humans and exhibit enhanced autoreactivity compared with their IgM^+/high^ counterparts.^22^, ^23^ Moreover, IgM^–/low^ mature naïve B cells carry longer HCDR3 sequences with more frequent J6 usage compared with IgM^+/high^ mature naïve B cells.^22^ These findings support a hypothesis where the IgM^low^ mature naïve B‐cell compartment serves as a repository of HCDR3 diversity in humans. Whether downregulation of surface IgM occurs in B cells bearing long HCDR3 as they enter the mature naïve stage or occurs earlier during peripheral B‐cell development is unknown. Therefore, we performed massively parallel sequencing of immunoglobulin heavy‐chain (IGH) repertoires in transitional and mature naïve peripheral blood B cells in the upper and lower quartiles of surface IgM expression. Our findings provide insights into the mechanisms that balance antibody repertoire diversity and self‐tolerance during peripheral B‐cell maturation.

## RESULTS

### Transitional and mature naïve B cells exhibit a broad dynamic range of surface IgM expression

**Figure 1:**
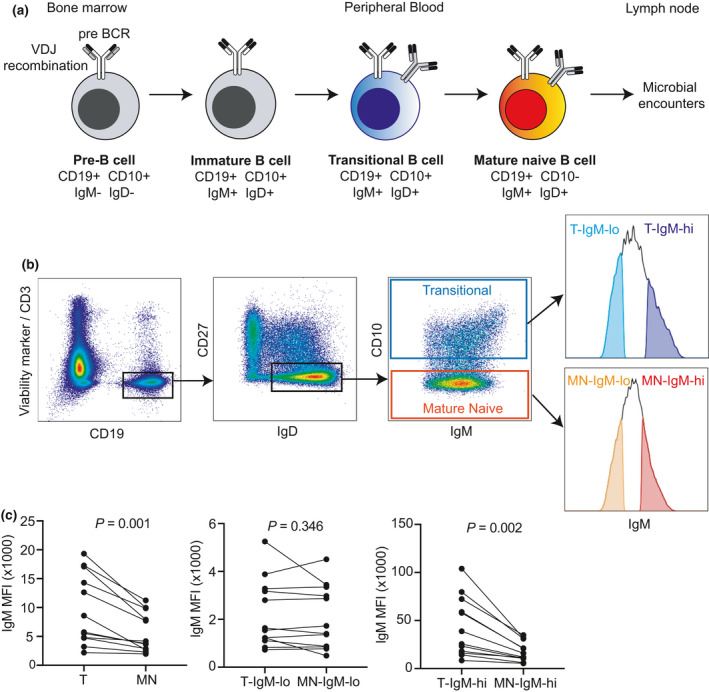
Flow cytometry gating strategy for sorting immunoglobulin M (IgM) high and low transitional and mature naïve (MN) B cells from healthy blood donors. **(a)** Schematic of B‐cell development illustrating expression of markers used to sort transitional and MN B cells in peripheral blood. **(b)** Representative flow cytometry gating strategy to obtain transitional and MN B cells within the upper and lower quartiles of surface IgM expression. Histograms demonstrating IgM^hi^ and IgM^lo^ populations for all donors are shown in Supplementary figure 1. **(c)** Surface IgM mean fluorescence intensity (MFI) for each cell population shown in **b**. Points represent IgM MFI for individual donors connected by solid line. *P*‐values for a paired *t*‐test comparing IgM MFI for paired data points of different cell populations from each donor.

To test the hypothesis that B cells with lowered surface IgM harbor increased HCDR3 diversity, transitional (CD19^+^CD27^−^IgD^+^CD10^+^) and mature naïve (CD19^+^CD27^−^IgD^+^CD10^−^) B cells within the upper and lower quartiles of surface IgM expression (sIgM^hi^ and sIgM^lo^, respectively) were flow sorted from 12 healthy donors (Figure 1a, b; Supplementary table 1). Each donor exhibited a broad dynamic range of IgM expression in both transitional and mature naïve B cell subsets (Supplementary figure 1). Transitional B cells had significantly higher surface IgM mean fluorescence intensity compared with mature naïve B cells (9619 ± 6146 *versus* 5677 ± 3415, *P* = 0.0013, paired *t*‐test). This difference was consistent when comparing transitional (T) and mature naïve (MN) B cells sorted from the surface IgM upper quartile (T‐sIgM^hi^ 43 102 ± 30 870 *versus* MN‐sIgM^hi^ 17 652 ± 10 233, *P* = 0.0017) but not in the IgM lower quartile (T‐sIgM^lo^ 2224 ± 1430 *versus* MN‐sIgM^lo^ 2051 ± 1319, *P* = 0.3463; Figure 1c). IGH sequencing was performed on 10 000 cells sorted from each quartile. After filtering, an average of 237 305.2 total reads were obtained per cell subset for each subject (range: 106 997–438 265), represented by 735 to 12 150 high‐quality, productive IgM IGH lineages per sample (mean: 5639.9; Supplementary figure 2). Two donors (142 and 809) consistently yielded lower clonal lineages across the four cell subsets compared with the other donors despite similar sequencing read depths.

### Transitional and mature naïve B cells with low surface IgM have longer HCDR3 than mature naïve B cells expressing high levels of surface IgM

**Figure 2:**
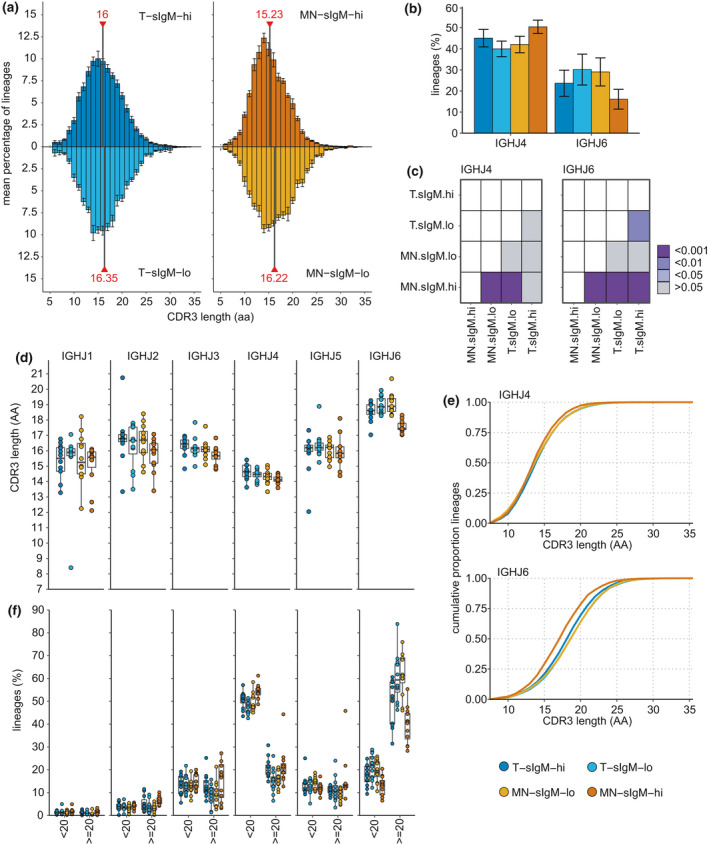
Third heavy‐chain complementarity‐determining region (HCDR3) length and IGHJ usage for transitional and mature naïve B cells within upper and lower quartiles of surface immunoglobulin M (IgM) expression. **(a)** Mean HCDR3 length distributions of 12 healthy donors for the four sorted populations. Surface IgM (sIgM)^high^ populations are plotted in upper part and sIgM^low^ in the lower part. Each bar indicates the mean percentage of B‐cell lineages from the subjects for a given CDR3 length [amino acid (AA) residues]. Error bars indicate the standard error of mean. Thick gray lines marked with red triangles indicate the overall mean CDR3 length for the 12 donors within a population. **(b)** Mean IGHJ4 and IGHJ6 usage for the 12 donors; error bars are 1 s.d. **(c)** *P*‐values for the Tukey honest significant difference ANOVA post‐test for differences in gene usage across the four sorted subsets for IGHJ4 and IGHJ6. **(d)** Mean CDR3 lengths for each subject for rearrangements using different IGHJs. Points show mean CDR3 length for each subject, boxplots show median and interquartile ranges. **(e)** Cumulative distribution of CDR3 lengths utilizing IGHJ4 and IGHJ6 across the 12 donors. Each line shows the cumulative proportion of lineages captured by increasing CDR3 lengths for each of the cell subsets. **(f)** Percentage of lineages using each of the IGHJ genes across the 12 subjects for CDR3s with fewer than 20 AA residues and those with 20 or more residues. Boxplots show median and interquartile ranges, points show percentage of lineages for each subject.

Longer HCDR3 sequence lengths can be advantageous for recognizing specific microbes but have also been associated with self‐reactive or polyreactive antibodies^2^, ^24^, ^25^, ^26^ and are enriched in out‐of‐frame IGH rearrangements, indicating a selection bias against longer HCDR3 length in human antibody repertoires.^15^ The mean HCDR3 lengths for the 12 donors follow an approximately normal distribution in each of the four cell populations (Figure 2a). In transitional B‐cell populations, HCDR3s from sIgM^lo^ B cells (16.35 ± 0.54, mean CDR3 AA residues ± s.d.) are not significantly longer than CDR3s from sIgM^hi^ B cells (16.00 ± 0.54, *P* > 0.05, Figure 2a). However, a shift in mean HCDR3 length is observed for the mature naïve B‐cell population in the upper *versus* lower quartile of sIgM expression. MN‐sIgM^hi^ B cells have significantly shorter HCDR3 lengths than the MN‐sIgM^lo^ B cells [15.23 ± 0.34 *versus* 16.22 ± 0.62, *P* = 0.00017, ANOVA Tukey honest significant difference (HSD) post‐test, Figure 2a]. Moreover, the MN‐sIgM^hi^ B‐cell CDR3 lengths are significantly shorter compared with the T‐sIgM^lo^ subset (*P* = 0.000023, ANOVA Tukey HSD post‐test).

### IGHJ6 rearrangements are negatively selected by the MN‐sIgM^hi^ population

Given that long HCDR3 sequences in the human B‐cell repertoire tend to be generated by long D and J gene segments in humans,^11^ we predicted skewed IGHJ gene usage in mature nature naïve B cells with low surface IgM compared with their high IgM counterparts. In the absence of exonuclease trimming, IGHJ6 contributes the highest number of IGHJ‐derived residues to the CDR3: 9 AAs compared with 4–6 AA residues for the other IGHJs.^27^ Comparison of IGHJ gene segment usage across the four subsets revealed that IGHJ6 was utilized on average at almost half the frequency in MN‐sIgM^hi^ (16.07 ± 4.70%, mean ± s.d.) compared with the MN‐sIgM^lo^ population (29.04 ± 6.66%, *P* = 4.77 × 10^−13^, ANOVA with Tukey HSD post‐test; Figure 2b, c, Table 1). IGHJ6 usage was also significantly lower in the MN‐sIgM^hi^ subset compared with the T‐sIgM^lo^ compartment (*P* = 3.35 × 10^−13^) which showed a similar IGHJ usage to the MN‐IgM^lo^ population (Figure 2a, b, *P* > 0.99, ANOVA with the Tukey HSD post‐test). The decrease in IGHJ6 utilizing IGHs among mature naïve B cells with high sIgM leads to a compensatory increase in the utilization of IGHJ4 (Figure 2b, Table 1). No difference in IGHJ segment usage frequencies was observed between T‐sIgM^lo^ and T‐sIgM^hi^ populations (all *P*‐values > 0.05, ANOVA with the Tukey HSD post‐test).

**Table 1:** Mean IGHJ frequencies ± s.d. among transitional and mature naïve B cells sorted for upper and lower quartile of surface IgM from 12 donors

Gene	T‐sIgM^hi^	T‐sIgM^lo^	MN‐sIgM^lo^	MN‐sIgM^hi^
*IGHJ1*	1.16% ± 0.67	1.44% ± 0.97	1.05% ± 0.66	1.78% ± 1.07
*IGHJ2*	3.99% ± 2.08	3.54% ± 1.76	3.14% ± 1.3	4.37% ± 1.17
*IGHJ3*	13.01% ± 4.1	12.52% ± 4.22	11.85% ± 3.69	15.01% ± 3.93
*IGHJ4*	45.06% ± 4.13	39.97% ± 3.7	42.07% ± 3.9	50.43% ± 3.18
*IGHJ5*	13.12% ± 3.26	12.38% ± 2.73	12.86% ± 2.47	12.49% ± 2.51
*IGHJ6*	23.66% ± 6.19	30.15% ± 7.34	29.04% ± 6.66	16.07% ± 4.7

IGHJ, immunoglobulin heavy‐chain joining; MN, mature naïve.

To determine whether biased IGHJ6 usage in recombination events is driving the observed differences in HCDR3 lengths, the mean number of HCDR3 residues were compared within each IGHJ rearrangement across the cell subsets. Rearrangements using IGHJ6 had longer mean CDR3 lengths compared with non‐IGHJ6 rearrangements in all four cell subsets (*P*‐values: MN‐sIgM^hi^ < 0.0001, MN‐sIgM^lo^ < 0.0001, T‐sIgM^hi^ < 0.0001, T‐sIgM^lo^ < 0.0001, pairwise Wilcoxon rank‐sum Bonferroni corrected), whereas IGHJ4 rearrangements had the shortest CDR3 lengths across the four subsets (Figure 2d). However, when HCDR3 sequences using the same IGHJ region were compared, the IGHJ6 CDR3 sequences were significantly shorter in MN‐sIgM^hi^ cells compared with all other subsets (17.58 AAs *versus* 18.53 T‐sIgM^hi^, 18.94 T‐sIgM^lo^, 19.03 MN‐sIgM^lo^, *P* = 0.003, <0.00001, <0.00001, respectively, pairwise Wilcoxon rank‐sum Bonferroni corrected test). The cumulative distribution of CDR3 lengths for IGHJ6 rearrangements reveals that approximately 25% of rearrangements have CDR3s that include more than 20 residues, whereas CDR3s of this length are almost absent from IGHJ4 rearrangements across the four subsets (Figure 2e). Among IGH with CDR3 lengths of 20 or more AAs, IGHJ6 is the dominant IGHJ compared with IGHJ4 dominance in IGH with CDR3 lengths under 20 AA residues (Figure 2f). On average, 60.53 and 61.77% of IGH with CDR3s exceeding 20 AA residues from the T and MN‐sIgM^lo^ populations, respectively, use IGHJ6, but for MN‐sIgM^hi^ cells this is reduced to 39.67%.

### Selection alters the characteristics of CDR3s in the sIgM^hi^ mature naïve compartment

**Figure 3:**
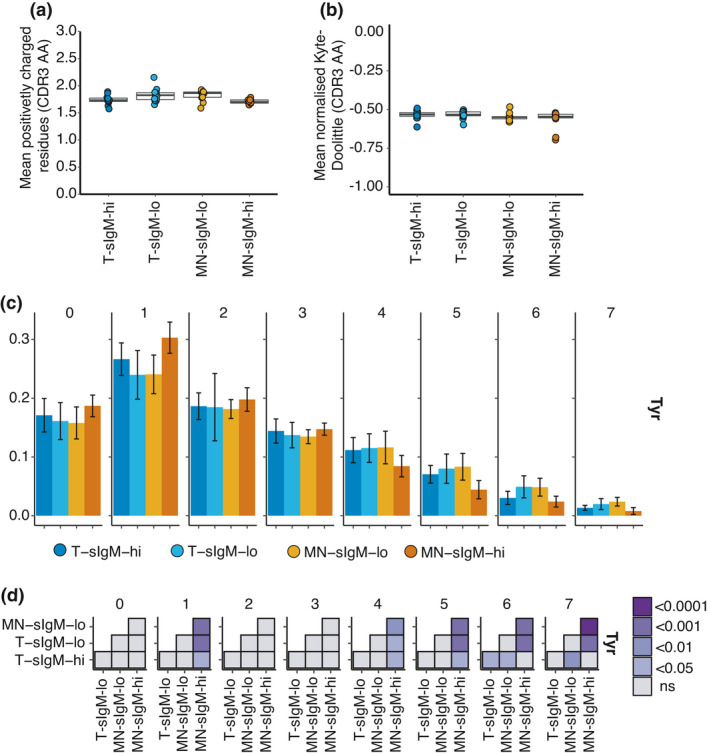
CDR3 properties of IGH from transitional and mature naïve B cells within the upper or lower quartiles of surface IgM expression. **(a)** Contribution of positively charged amino acids to third heavy‐chain complementarity‐determining region (HCDR3) for sorted B‐cell populations. Points show the mean number of positively charged residues for each subject, and boxplots show median and interquartile ranges. **(b)** Length‐normalized Kyte–Doolittle scores for the hydrophobicity of the CDR3 residues for sorted B‐cell populations for 12 subjects. **(c)** Each subpanel shows the frequency of CDR3s with the indicated number of tyrosine residues ranging from 0 to 7 residues. Each bar plots the mean frequency of IGH chains with the indicated number of CDR3 tyrosine across the 12 subjects with error bars showing s.d. **(d)** *P*‐values from ANOVA with a Tukey honest square distance post‐test to test significance across the subsets for the mean frequency of different tyrosine counts within the CDR3s. Panels show results for tyrosine counts ranging from 0 to 7 and *P*‐value is indicated by the color scale. IGH, immunoglobulin heavy‐chain.

In addition to length, other CDR3 properties may influence B‐cell selection, for example, B‐cell receptors (BCRs) with highly positively charged CDR3s are more self‐reactive^2^ and hydrophobic CDR3s are negatively selected during B‐cell maturation.^15^ In the current study, a small decrease in the number of positively charged CDR3 residues was observed for the sIgM^hi^ compared with the sIgM^lo^ subsets (Figure 3a, *P* = 0.014 MN‐sIgM^hi^ *versus* T‐sIgM^lo^, ANOVA with Tukey HSD post‐test). However, no difference was observed between any of the four subsets for the number of negatively charged CDR3 residues (all *P*‐values > 0.05). There were no differences in the mean hydrophobicity of the CDR3 residues between the sorted subsets following normalization for CDR3 length (Figure 3b, all *P* > 0.05). These results suggest that the MN‐sIgM^hi^ compartment is subject to differing selection biases than MN B cells that have downregulated their sIgM for both CDR3 length and AA composition. There were differences in the proportion of specific AAs contributing to the CDR3s in IgM^hi^ and IgM^lo^ subsets with increased Ser, Thr, Asn, Ala and His and decreased Tyr, Trp and Leu in IgM^hi^ compared with IgM^lo^ CDR3s (Supplementary figure 3). Of note was the selection against tyrosine (Y) residues within the MN‐sIgM^hi^ compartment. This may be driven by the decreased use of full‐length IGHJ6 in the MN‐sIgM^hi^ compartment, as full‐length IGHJ6 contributes YYYYYGMDV to the CDR3. When considering the number of tyrosine residues within the CDR3 there was a selection against four or more tyrosine residues for the MN‐sIgM^hi^ compared with the other three subsets (Figure 3c, d) with a significant increase for CDR3s that include only a single tyrosine for the MN‐sIgM^hi^. Other AAs also had distinct usage among the different cell subsets, and these are plotted in Supplementary figure 4.

Tyrosine residues may undergo post‐translational modifications including sulfation, which occur at particular motifs^28^, ^29^ and which have been linked to altered antibody function.^30^, ^31^ The frequency of sulfation (Supplementary figure 5) motifs among CDR3s with varying number of tyrosine residues across the different cell subsets was determined and the motif frequency was unchanged across the populations.

### Selection that impacts CDR3 length begins prior to transitional B‐cell stage

**Figure 4:**
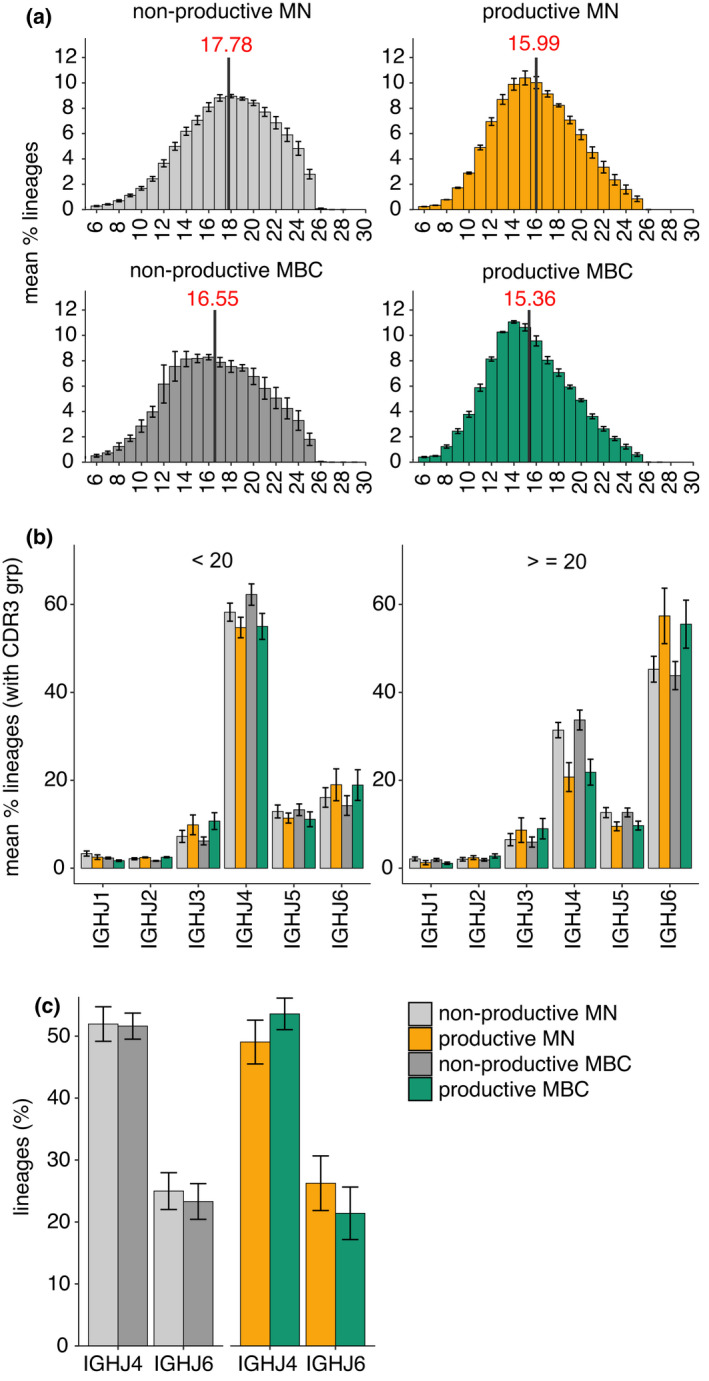
CDR3 lengths and IGHJ usage in productive and nonproductive IGH from total mature naïve (MN) and memory B‐cell (MBC) populations. **(a)** CDR3 length distributions for productively and nonproductively rearranged IGH from three donors sequenced by Adaptive Biotechnologies.^32^ Bars show mean percentage of B‐cell lineages for each CDR3 length; error bars are standard error of mean. The solid gray line is the mean CDR3 length for each population. **(b)** IGHJ gene segment usage for productive and nonproductive rearrangements from MN and MBCs. Bars are mean percentage of lineages utilizing each IGHJ ± standard error of mean. CDR3s are segregated by those with fewer than 20 amino acids (AAs) and those with 20 or more AA residues. **(c)** Mean percentage of lineages using either IGHJ4 or IGHJ6 (±standard error of mean) for sorted cell subsets. IGHJ, immunoglobulin heavy‐chain joining.

It is clear that selection occurring between the transitional and mature naïve alters the composition of the BCR repertoire, and that this selection is most impactful on the mature naïve cells with the highest surface IgM expression. To explore how much selection alters the repertoire prior to the transitional B‐cell stage, we examined nonproductive rearrangements from three donors from a published data set of MN and memory B cells.^32^ These nonproductive rearrangements were not subject to selection as the B cells that carry them were selected based on the expressed BCR rearrangement on the other chromosome. The mean nonproductive CDR3 length of MN cells from the published data set was 17.78 AAs (Figure 4a), which is longer than the means for the transitional sIgM high and low populations determined in this study (16.00 and 16.35, respectively; Figure 2a). These data suggest that the CDR3 length has already been selected toward shorter regions prior to the transitional B‐cell stage. Interestingly, among the nonproductive rearrangements with 20 or more AAs, the skew toward IGHJ6 usage is less than for the productive rearrangements (Figure 4b). Long CDR3s for nonproductive rearrangements are approximately 30% IGHJ4 and approximately 40% IGHJ6 compared with approximately 20% and approximately 60%, respectively, for productive rearrangements, suggesting that length alone does not drive the selection. The IGHJ gene usage among the productive and nonproductive total MN and memory B cell populations suggests that the strong selection against IGHJ6 is unique to the sIgM^hi^ MN cells (Figure 4c); however, the productive memory B cells possess CDR3 lengths (15.36 AA) and IGHJ usage that are consistent with the sIgM^hi^ MN that could indicate that these sIgM^hi^ subsets are more likely to become memory B cell than their sIgM^lo^ counterparts.

### IgM^hi^ and IgM^lo^ subsets do not have distinct IGHV gene usage

**Figure 5:**
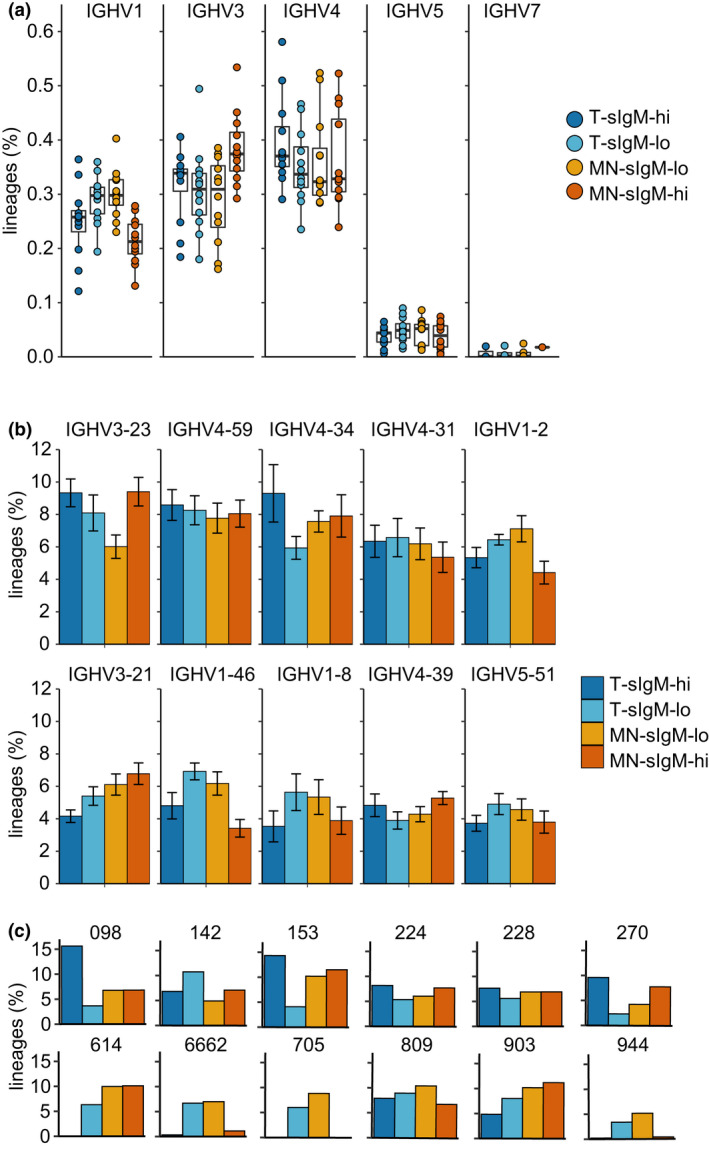
IGHV gene usage for transitional and mature naïve B‐cell compartments for cells with high or low surface IgM. **(a)** IGHV family utilization across the cell subsets. Boxplots show median and interquartile ranges. Data points for each subject are plotted. **(b)** IGHV4‐34 frequency in the four sorted cell subsets for 12 subjects. **(c)** Percentage of total B‐cell clones using each IGHV gene segment across the four sorted cell subsets for the top 10 IGHVs (see Supplementary figure 6 for all IGHVs). Bars show mean usage across the 12 donors and error bars indicate 1 s.d. IGHV, IGHJ, immunoglobulin heavy‐chain variable.

While HCDR3 imparts the most diversity to the antibody repertoire, the large number (> 40) of functional IGHV gene segments available for the human repertoire creates significant breadth. IGHV gene usage is not entirely random with certain regions used recurrently while others are rare.^33^ To determine whether IgM^lo^ subsets serve as a repository for diverse IGHV genes, harboring rare or different IGHV gene segments compared with their IgM^hi^ counterparts, we evaluated IGHV usage across transitional and mature naïve B cells in the upper and lower quartiles of surface IgM expression (Supplementary figure 6). At the IGHV family level, there were trends toward increased IGHV1 usage among sIgM^lo^ populations relative to sIgM^hi^ populations and the MN‐sIgM^hi^ subset was enriched for IGHV3 rearrangements compared with the other subsets (Figure 5a, ANOVA with Tukey HSD post, all *P* > 0.05). Overall, rearrangements using 49 different IGHV segments were observed across the 12 healthy donors. Rearrangement frequencies for the top 10 most commonly utilized segments are plotted in Figure 5b. On average, the most frequently utilized IGHV segment within the T and MN‐sIgM^hi^ subsets was IGHV3‐23 (9.33 and 9.40%, respectively), consistent with previously reported peripheral MN B‐cell repertoires.^33^, ^34^ Within the T and MN‐sIgM^lo^ subsets, IGHV4‐59 was on average the most frequently utilized IGHV (8.26 and 7.77%, respectively). When considering mean IGHV utilization across the 12 donors, IGHV3‐23, IGHV4‐34 and IGHV1‐46 were most differentially utilized across the cell subsets (Figure 1a, ANOVA and the Tukey HSD post‐test). IGHV3‐23 usage within the MN‐sIgM^lo^ was significantly lower than for the T sIgM^hi^ (*P* = 0.0069) and MN‐sIgM^hi^ (*P* = 0.0041) compartments and IGHV4‐34 utilization was higher among the T sIgM^hi^ compared with the T sIgM^lo^ population (*P* = 0.0050). IGHV1‐46 trended toward higher usage among sIgM^lo^ compartments compared with sIgM^hi^ and was significantly reduced among the MN‐sIgM^hi^ compared with the T sIgM^lo^ population (*P* = 0.0017).

Individual donors vary in their utilization of the different gene segments. Each donor was therefore analyzed independently, and all donors had statistically significant associations between IGHV gene utilization frequencies and the four sorted cell subsets (Chi‐squared test, all *P*‐values < 2.2 × 10^−16^). Analysis of the Pearson residuals (Supplementary figure 7a) and the percentage contribution of the residuals to the overall Chi‐squared statistics (Supplementary figure 7b) indicate that the gene segments that contribute to the difference vary across the individuals. IGHV4‐34 contributed to the differences between the four subsets in many donors; for donors 944, 705 and 6662 much of the dependency between IGHV gene and cell subset was driven by differences in the frequency of IGHV4‐34 across the different cell subsets, where IGHV4‐34 was virtually absent in sIgM^hi^ subsets and positively associated with the sIgM^lo^ MN and T compartments (Figure 5c). This is consistent with IGHV4‐34 encoding an innately self‐reactive IGHV region with specificity for I/i antigen on erythrocytes and B cells^35^, ^36^ and being confined to B cells with lower surface IgM.^37^

### B‐cell developmental markers and surface IgM expression

Levels of IgM expressed on the B‐cell surface have been correlated with B‐cell maturity and autoreactivity.^21^, ^38^, ^39^, ^40^ Transitional B cells had, on average, higher surface expression of IgM than circulating mature naïve B cells (Figure 1c). Therefore, it is possible that downregulation of IgM represents a developmental process and the IgM^lo^ B‐cell subsets studied herein reflect more mature B cells compared with their IgM^hi^ counterparts. To evaluate B‐cell maturity in IgM^lo^ and IgM^hi^ transitional subsets, we compared surface CD10 expression, which gradually decreases on transitional B cells upon exiting the bone marrow.^41^ CD10 expression was significantly lower on T IgM^lo^ compared with T IgM^hi^ B cells (mean fluorescence intensity 1743 ± 1284 *versus* 2929 ± 2810, *P* = 0.0282, paired *t*‐test; Supplementary figure 8a), suggesting that T IgM^lo^ B cells are more mature than their T IgM^hi^ counterparts. In mature naïve subsets we evaluated surface IgD expression, which tends to be maintained or increased on autoreactive B cells that downregulate IgM in response to self‐antigen.^21^, ^22^, ^23^, ^37^, ^38^, ^42^ In this study, transitional B cells had significantly higher IgD surface expression compared with mature naïve B cells (mean fluorescence intensity 4431 ± 2860 *versus* 3615 ± 2041, *P* = 0.0163, paired *t*‐test). Moreover, surface IgD expression correlated with IgM, where IgM^lo^ B cells had significantly lower surface IgD compared with IgM^hi^ B cells for both transitional (T‐sIgM^lo^ 3001 ± 1978 *versus* T‐sIgM^hi^, 5892 ± 3779 *P* = 0.0008) and mature naïve compartments (MN‐sIgM^lo^ 2,509 ± 1,435 *versus* MN‐sIgM^hi^, 4874 ± 2571 *P* < 0.0001) (Supplementary figure 8b).

## DISCUSSION

The ability to generate a diverse antibody repertoire is essential for host protection from a broad array of viruses and bacteria with different species utilizing a multitude of tactics to preserve antibody breadth. Here we report a strategy in humans for maintaining BCRs with “permissive” heavy‐chain CDR3 properties, including longer sequence lengths and increased IGHJ6 and aromatic AA usage, in the circulating mature naïve compartment in the lowest quartile of surface IgM expression. These findings support the idea that IgM^lo^ B cells serve as a repository of antibody diversity by maintaining a breadth of receptors, including those with properties associated with self‐reactivity.^2^, ^15^, ^24^, ^25^, ^26^

Mature naïve B cells with IGH rearrangements that include IGHJ6 are more likely to be self‐reactive than their counterparts using other IGHJ segments.^22^ Therefore, selection against IGHJ6 in mature naïve cells with high surface IgM expression may result from two nonmutually exclusive explanations. Self‐reactive B cells expressing IGHJ6 may survive a transitional to mature naïve checkpoint by decreasing the expression of surface IgM and becoming anergic, thereby resulting in an increase in mature naïve B cells with low surface IgM using IGHJ6. In addition, transitional B cells that include IGHJ6 and fail to downregulate surface IgM upon becoming mature naïve may be lost at this checkpoint by apoptotic deletion, hence reducing the number of IGHJ6 mature naïve B cells with high surface IgM. Our current data set is not able to distinguish whether either or both of these possibilities occur. Because IGHJ6 contributes more residues to the CDR3 than other IGHJ segments, we surmised that the increased CDR3 length observed in surface IgM^low^ mature naïve B cells was a result of increased IGHJ6 usage. However, comparison of mature naïve B cells with IGHJ6 revealed shorter CDR3 lengths in IgM^high^ cells compared with IgM^low^ cells, suggesting that the selection is against IGHs that have rearranged IGHJ6 with minimal trimming of nucleotides from the gene segment end during DJ recombination. As a corollary, IGHJ6 rearrangements in possession of shorter CDR3 lengths may have altered physiochemical properties that permit their maintenance in the mature naïve sIgM^hi^ pool. The selection against aromatic residues (proline, tyrosine, tryptophan) in the IgM^high^ mature naïve population may contribute to altering physiochemical properties favorable to mature naïve IgM^hi^ B cells. Moreover, the IgM^high^ mature naïve B cells using IGHJ4 had significantly shorter mean CDR3 lengths than their IgM^low^ counterparts. These data suggest that a selection checkpoint from transitional B cells to mature naïve B cells not only decreases IGHJ6 segment usage, but also decreases the CDR3 length.

Levels of surface IgM exhibit a broad dynamic range and are influenced by multiple factors including stage of development and stimulation by antigen. The expression of IgM and IgD constant region exons (Cμ and Cδ, respectively) is coordinately regulated at the transcriptional level by alternative splicing of a long primary messenger RNA transcript containing V(D)J exons, Cμ and Cδ. Developing B cells exclusively express IgM and upon emerging from the bone marrow, coexpress IgM and IgD. Mature follicular B cells contain more Cμ messenger RNA than Cδ; however, IgD is expressed at a higher density on the B‐cell surface because of increased stability and translational efficiency of Cδ transcripts and preferential binding of IgD over IgM to CD79α/β BCR assembly subunits.^43^, ^44^, ^45^ Surface IgM expression is more sensitive to antigen‐dependent fluctuation than IgD, and therefore, self‐reactive B cells tend to display low surface IgM while maintaining high surface IgD expression.^21^, ^22^, ^23^, ^37^, ^38^, ^42^ By contrast, the B cells in the lowest quartile of surface IgM expression studied herein also had significantly lower surface IgD expression compared with B cells in the highest quartile of IgM expression. The IgM^lo^ MN B cells may be more representative of transgenic BCRs reactive with nuclear autoantigens that exhibited progressive downregulation of surface IgM and IgD during development in the bone marrow and periphery in a process termed “learned ignorance.”^46^ In this model these transgenic IgM^lo^/IgD^lo^ B cells were activated by polyclonal stimuli, participated in germinal center reactions and were not short lived. It was proposed that progressive downregulation of both IgM and IgD reduced cellular avidity for autoantigens, which may enable autoreactive B cells to participate in immune responses to foreign antigens.^46^ While it is unknown whether the IgM^lo^ MN B cells reported here have undergone a similar process of progressively reducing surface BCR expression, it is plausible that these cells harboring diverse and potentially autoreactive HCDR3 sequences are poised for germinal center recruitment where somatic hypermutation can attenuate affinity for self‐antigens.^18^, ^19^, ^20^

When considering all 12 donors, there was no significant difference in IGHV segment usage in peripheral blood B cells grouped according to surface IgM expression. This may have been driven by individual variation in gene usage frequencies for the different donors. For example, B cells using unmutated IGHV4‐34 gene segments are potentially self‐reactive and are generally of an anergic B‐cell phenotype with downregulated surface IgM.^37^ Therefore, it was somewhat unexpected that only 3 of 12 patients demonstrated an enrichment of IGHV4‐34 B cells in IgM^low^ subsets. The use of an anti‐idiotypic antibody, 9G4, that binds IGHV4‐34 B cells in a subset of patients revealed that over 90% of IGHV4‐34 B cells are absent from the upper and lower quartiles of IgM expression (data not shown). These findings suggest that HCDR3 length and composition exert more selection pressure on B cells than any given IGHV segment, even those that are inherently self‐reactive such as IGHV4‐34.

Overall, our data identified the IGH repertoire properties that define transitional and mature naïve B cells within the upper and lower quartiles of surface IgM expression. Mature naïve B cells with low surface IgM exhibit features consistent with self‐reactive BCRs including increased usage of IGHJ6, longer HCDR3 lengths and increased aromatic residues compared with their IgM^high^ counterparts. These differences illuminate a repository of HCDR3 breadth that may serve to maintain antibody diversity and prevent holes in the primary immune repertoire.

## METHODS

### Cell isolation and sorting

Blood samples from healthy donors were obtained from the Australian Red Cross Blood Service. Peripheral blood mononuclear cells were prepared by Ficoll Histopaque (GE Healthcare, Chicago, IL, USA) and cells were cryopreserved in 50% fetal bovine serum, 40% RPMI (Thermo Fisher Scientific, Boston, MA, USA) and 10% dimethyl sulfoxide for 10–100 million/tube. Nearly 50–100 million peripheral blood mononuclear cells were thawed and washed in RPMI medium. Cells were then incubated with Fc block (BioLegend, San Diego, CA, USA) for 30 min at 4°C. After washing, cells were stained with antibodies against CD19 (BV421, HIB19; BioLegend), IgD (PerCP‐CY5.5, IA6‐2; BD, San Diego, CA, USA), CD27 (PE‐CY7, M‐T271; BD), CD10 (PE, HI10a; BD), IgM (BV421, MHM‐88; BioLegend), CD3 (APC‐CY7, SK7; BD), CD14 (APC‐CY7, MOP9; BD) and viability dye (eFluor780; eBioscience). Transitional B cells (CD19^+^CD27^−^IgD^+^CD10^+^) with IgM^hi^ (top 25%) and IgM^lo^ (bottom 25%), and mature naïve B cells (CD19^+^CD27^−^IgD^+^CD10^+^) with IgM^hi^ (top 25%) and IgM^lo^ (bottom 25%) were sorted on BD FACS Aria Ⅲ into 350 µL RLT lysis buffer (Qiagen, Venlo, Netherlands) and frozen at –80°C. For each donor, 10 000 cells were sorted for each population.

### BCR repertoire sequencing from sorted B‐cell subsets

RNA was extracted by using the RNeasy Micro Kit (Qiagen) and reverse transcribed to complementary DNA using the Smart‐seq2 protocol.^47^ Briefly, 2.25 µL RNA was used for reverse transcription and PCR ampliﬁcation as described with the exception of reducing the IS PCR primer to a 50 nM ﬁnal concentration and increasing the number of PCR cycles to 28. To amplify the IGH BCR transcripts from the complementary DNA, a two‐round PCR protocol was used. The first round used a multiplex PCR with gene‐specific V‐forward primers and an IgM‐reverse primer specific for the first exon of the IgM constant region (CH1; Supplementary table 1). PCRs were performed in a total volume of 25 µL containing 5 µL complementary DNA, 10 nM of each primer and 12.5 µL Q5 Master Mix (New England Biolabs, Ipswich, MA, USA). The PCR condition was set as 98°C for 30 s, followed by 40 cycles of 98°C for 10 s, 67°C for 30 s, 72°C for 40 s, with a final extension for 2 min at 72°C. PCR products were visualized on a 1% agarose gel and bands were excised and purified using the Qiagen MinElute Gel Extraction Kit. The second round PCR was performed to add indexes to each sample (Nextera XT Index Kit; Illumina, San Diego, CA, USA). The PCR conditions were 98°C for 30 s, followed by five cycles of 98°C for 10 s, 63°C for 30 s and 72°C for 30 min. Finally, the PCR products were purified by AMPure XP beads (Beckman Coulter, Brea, CA, USA) to remove primer sequences. Equimolar PCR products were pooled and sequenced on an Illumina MiSeq reagent kit v3 to generated 2 × 300 paired end reads.

### Processing of BCR repertoire sequence data sets

Illumina sequencing libraries were demultiplexed using the index reads. Paired end reads from FASTQ files were then merged using FLASH,^48^ converted to FASTA using the FASTX‐Toolkit [http://hannonlab.cshl.edu/fastx_toolkit] and trimmed of forward and reverse primers. Reads lacking an exact, full‐length match to primers were discarded. IgM isotype amplifications were confirmed by exact, full‐length matches to the portion of the IgM CH1 exon upstream from the reverse primer site (IgM‐CH1‐exon: GGGAGTGCATCCGCCCCAACCCTTTTCCCCC).

Primer‐trimmed reads were processed using stand‐alone IgBLAST^49^ with the IMGT human IGH variable (IGHV), diversity (IGHD) and joining (IGHJ) reference directories^50^ to determine which IGH gene segments comprised each rearrangement. IgBLAST output was also used to call mismatches to germline and to delineate CDRs and framework regions. IgBLAST parameters were default with the exception of returning only a single alignment for each gene segment.

Sequences that did not meet the following criteria were discarded from analysis: an IGHV gene segment of at least 270 nucleotides, the IGHV family matched to the forward primer (to remove hybrid templates arising from mispriming by incomplete extension from prior cycles in subsequent PCR cycles), an identifiable IGHJ, an unmutated IGHV as expected for transition and naïve compartments (> 98% identity to germline genes to allow for unreported allelic variants that were not in the IMGT reference) and a CDR3 (IMGT nomenclature) of at least five AA residues. Productive rearrangements were defined as those that lacked stop codons and were in‐frame for the IGHJ gene segment.

To infer sequences derived from the same B‐cell clone, or sequence variants that may have arisen because of PCR or sequencing error, sequences were clustered based on the CDR3 nucleotide sequence using cd‐hit‐est.^51^ Clustering was undertaken on sets of CDR3 nucleotide sequences from rearrangements that shared the same combination of IGHV (ignoring the allele), IGHJ (ignoring the allele) and CDR3 length. Clustering was performed using a 90% identity threshold.

For each cluster within a sample, the most numerous CDR3 AA sequence was selected as representative. CDR3 sequence metrics including net charge and counts for the number of positive, negative, aromatic and basic AA residues were calculated using the representative CDR3 AA sequence.

To establish IGHJ usage frequencies prior to selection, mature naïve (CD19^+^CD27^–^IgM^+^) and memory B cell (CD19^+^CD27^+^) repertoires sequenced from genomic DNA for three subjects generated by Adaptive Biotechnologies for DeWitt and colleagues^32^ were analyzed. The genomic DNA‐amplified rearrangements capture nonproductive rearrangements that have not been subjected to negative selection at a much higher frequency than the transcript libraries. Sequence data were obtained from Adaptive Biotechnologies (Seattle, WA, USA)^32^ and processed in the same manner as our data sets with the exception of retaining nonproductive rearrangements, requiring a minimum IGHV length of 50 nucleotides (as a result of the data only being about 130‐bp amplicons) and not filtering based on primer matches as the data sets were already primer trimmed.

### Data analysis of IGH repertoires

Analysis of IGH repertoires was undertaken at the B‐cell clone level within each sample. Only clones supported by more than a single read were included in analysis. This prevented overcounting of reads that were increased in copy number from the PCR and also lessened impact of sequence variants that had arisen because of PCR or sequencing error.

Analysis was undertaken in RStudio (Boston, MA, USA). Statistical significance for metrics across the different cell subsets such as gene usage or CDR3 length was determined using ANOVA followed by a Tukey HSD post‐test or pairwise Wilcoxon rank‐sum tests corrected for multiple hypothesis testing using Bonferroni correction.

Tyrosine post‐translation modification motifs were predicted for each clonal lineage‐dominant CDR3 using the GPS‐TSP tool for tyrosine sulfation.^52^ GPS‐TSP was run in batch mode with the prediction threshold set to “high.”

## CONFLICT OF INTEREST

The authors declare no conflict of interest.

## AUTHOR CONTRIBUTIONS

**Zhiguo Zhang:** Data curation; Formal analysis; Funding acquisition; Investigation; Writing‐original draft. **Christopher J** **Jara:** Investigation. **Mandeep Singh:** Investigation. **Huji Xu:** Funding acquisition. **Christopher C** **Goodnow:** Conceptualization. **Katherine JL Jackson:** Conceptualization; Data curation; Formal analysis; Funding acquisition; Supervision; Writing‐original draft; Writing‐review & editing. **Joanne H Reed:** Conceptualization; Formal analysis; Funding acquisition; Project administration; Supervision; Writing‐original draft; Writing‐review & editing.

## DATA AVAILABILITY STATEMENT

The data that support the findings of this study are openly available in Sequence Read Archive (SRA) at https://www.ncbi.nlm.nih.gov/sra, reference number BioProject PRJNA728806.
