# Supplementary material for: Human transitional and IgMlow mature naïve B cells preserve permissive B‐cell receptors
Source: Immunol Cell Biol. 2021 Jun 4;99(8):865–78. doi: 10.1111/imcb.12478 (PMC8453828; doi:10.1111/imcb.12478)
Supplement: Supplementary file 1 [file IMCB-99-865-s001.pdf]

Supplementary Table 1 Age, sex and IgM surface expression (mean fluorescence intensity, MFI) for sorted B cell populations from 12 healthy donors

Donor ID	Sex	Age (years)	Surface IgM MFI					
			transitional	T-IgM hi	T-IgM lo	mature naïve	MN-IgM hi	MN-IgM lo
98	M	75	12637	58914	3174	7720	20808	2977
142	F	56	3212	14129	821	2271	6003	851
153	M	56	5537	16056	1226	3752	11608	1365
224	F	na	4794	18110	1541	4181	10586	1725
228	M	33	17089	57891	5250	7907	21132	3442
270	M	73	19319	103978	3280	9972	31746	3352
614	M	45	5733	23354	1638	3654	10723	1395
705	M	57	17276	79736	3883	11248	31025	4507
809	F	25	2205	8400	726	1979	5394	770
903	M	71	14298	72288	2803	9870	34871	2872
944	F	47	4733	25617	1113	2888	11940	870
6662	F	na	8598	38756	1228	2680	15986	481

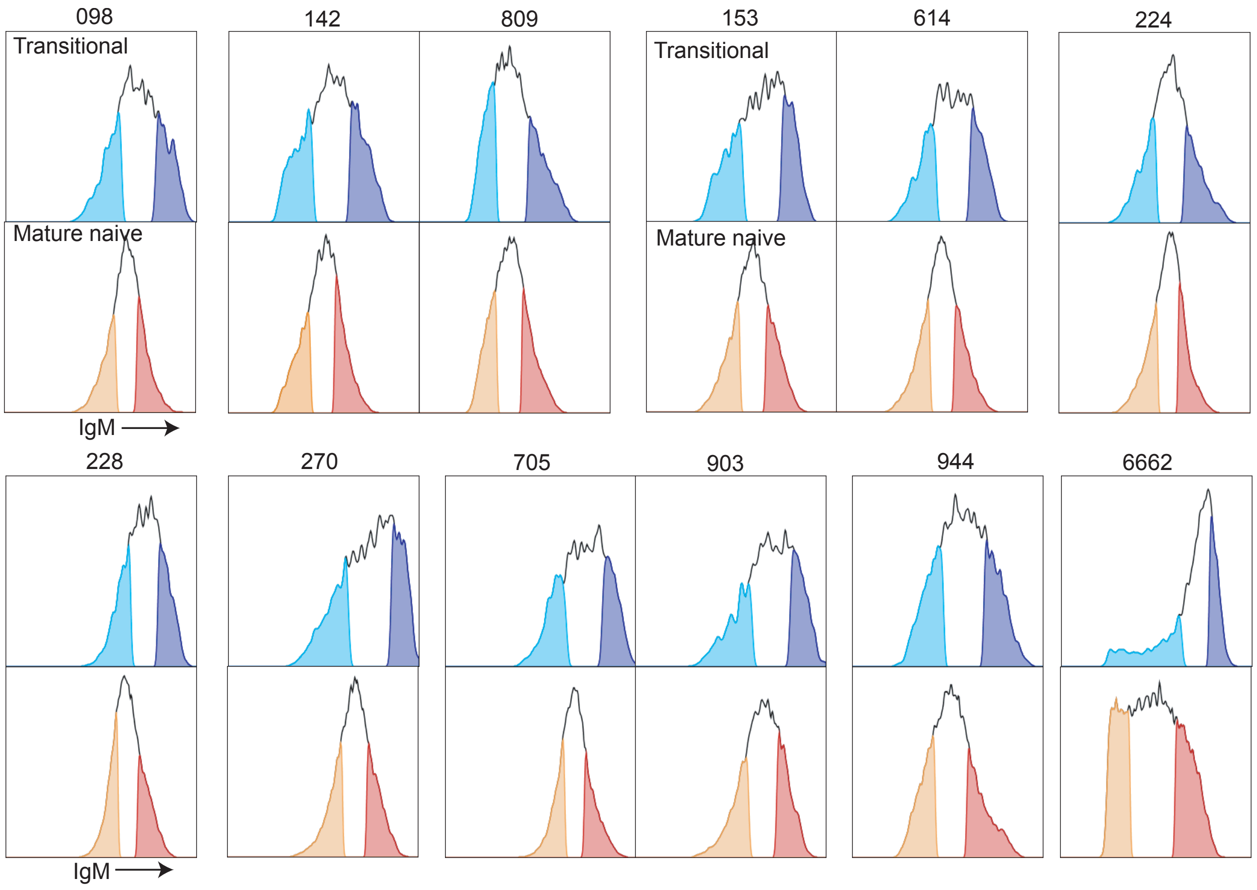

Supplementary Figure 1 Surface IgM expression for 12 healthy donors. IgM mean fluorescence intensity is shown for transitional and mature naïve B cells with upper and lower quartiles shaded. Numbers correspond to donor ID in Supplementary Table 1. Histograms are arranged according to experiment, where donors run in the same experiment are grouped.

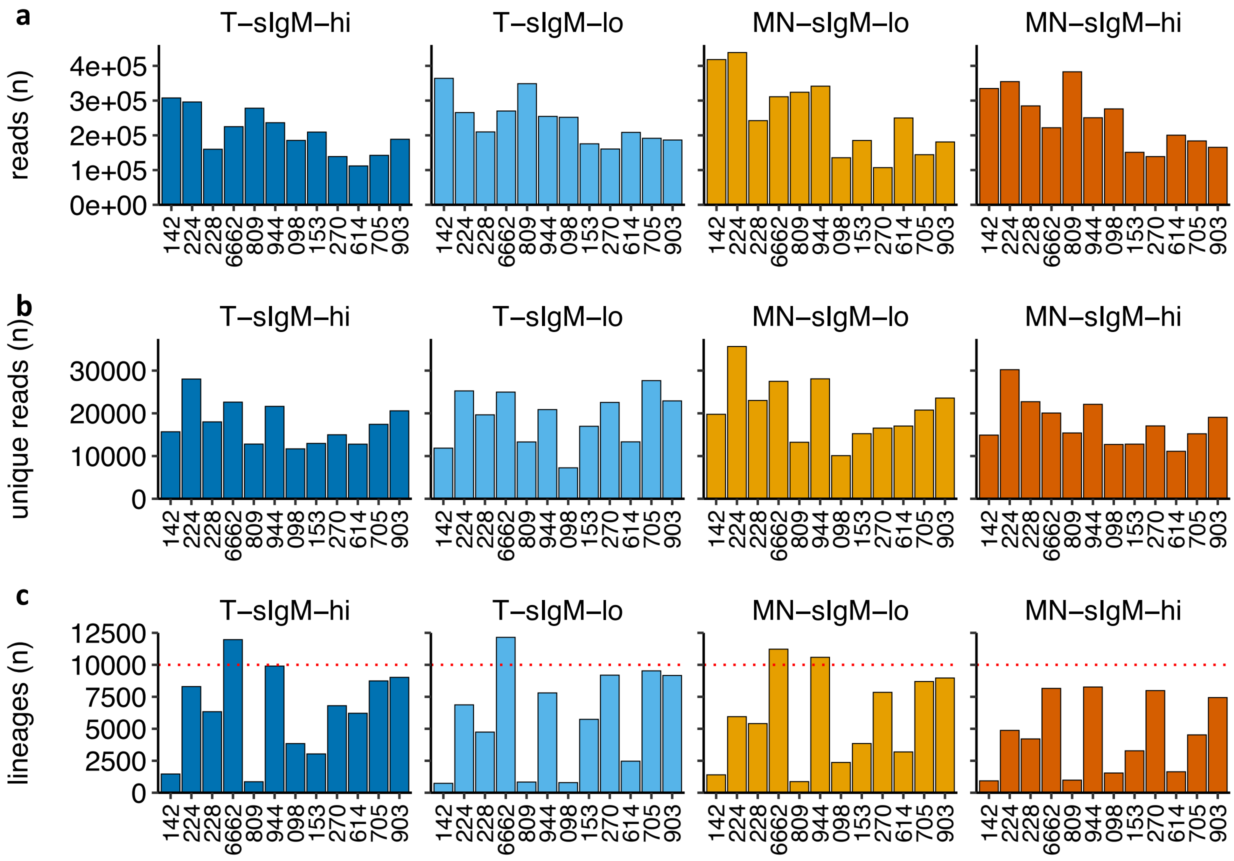

Supplementary Figure 2 Metrics for deep sequencing of IGH from sorted transitional (T) and mature naïve (MN) B cell populations from the upper (hi) and lower (lo) quartiles of surface (s) IgM expression. (**a**) Total sequencing reads for each sample. (**b**) Number of unique reads following deduplication (collapsing of identical reads to a single sequence). (**c**) Number of clonal lineages used for analysis of each sample.

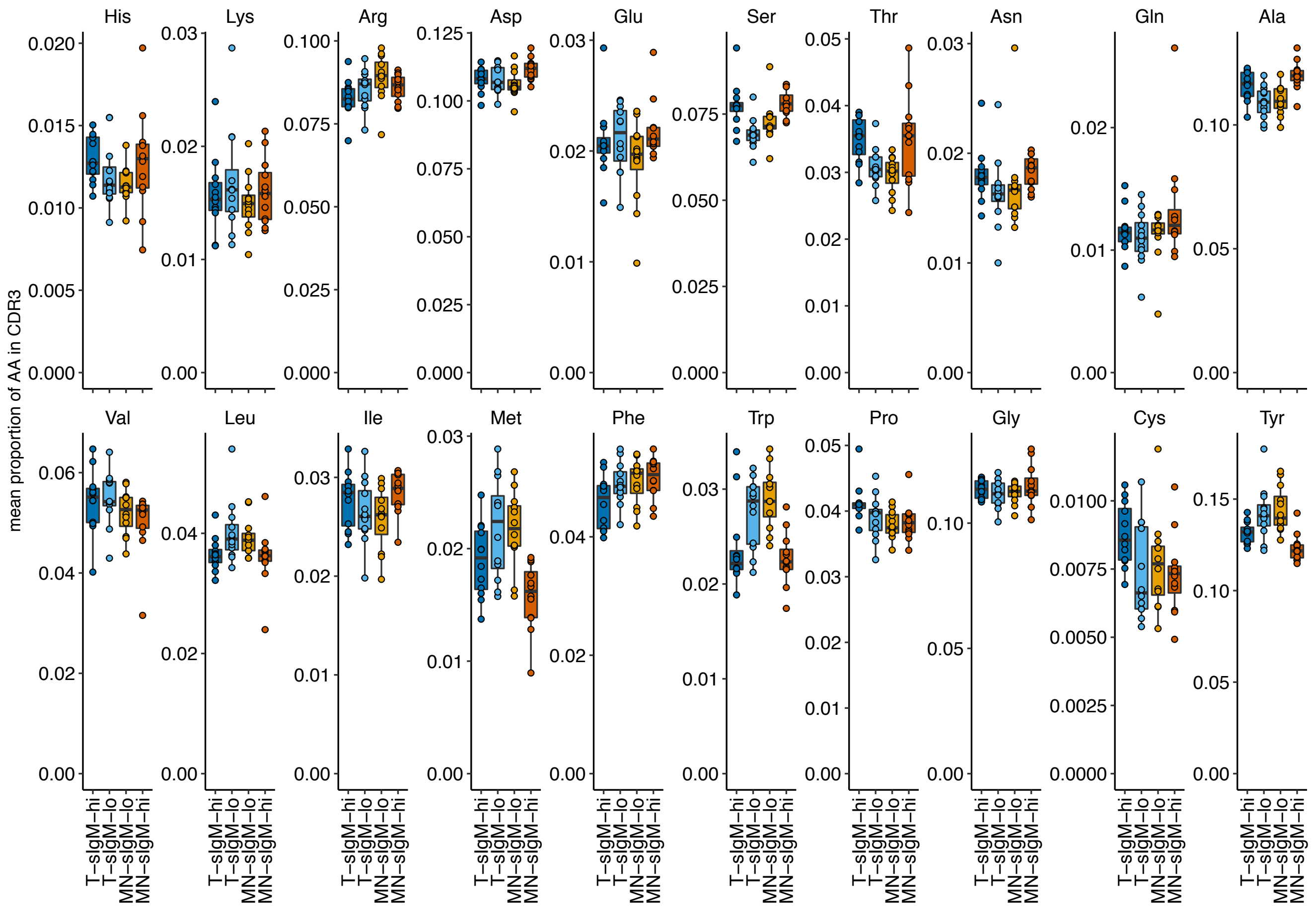

Supplementary Figure 3 Mean usage of amino acids in CDR3s from each cell subset. Each sub-panel plots the mean utilisation of an amino acid among the CDR3s for the clonal lineages for each donor. Boxplots summarise median and interquartile ranges. Data points show mean amino acid proportion for each donor.

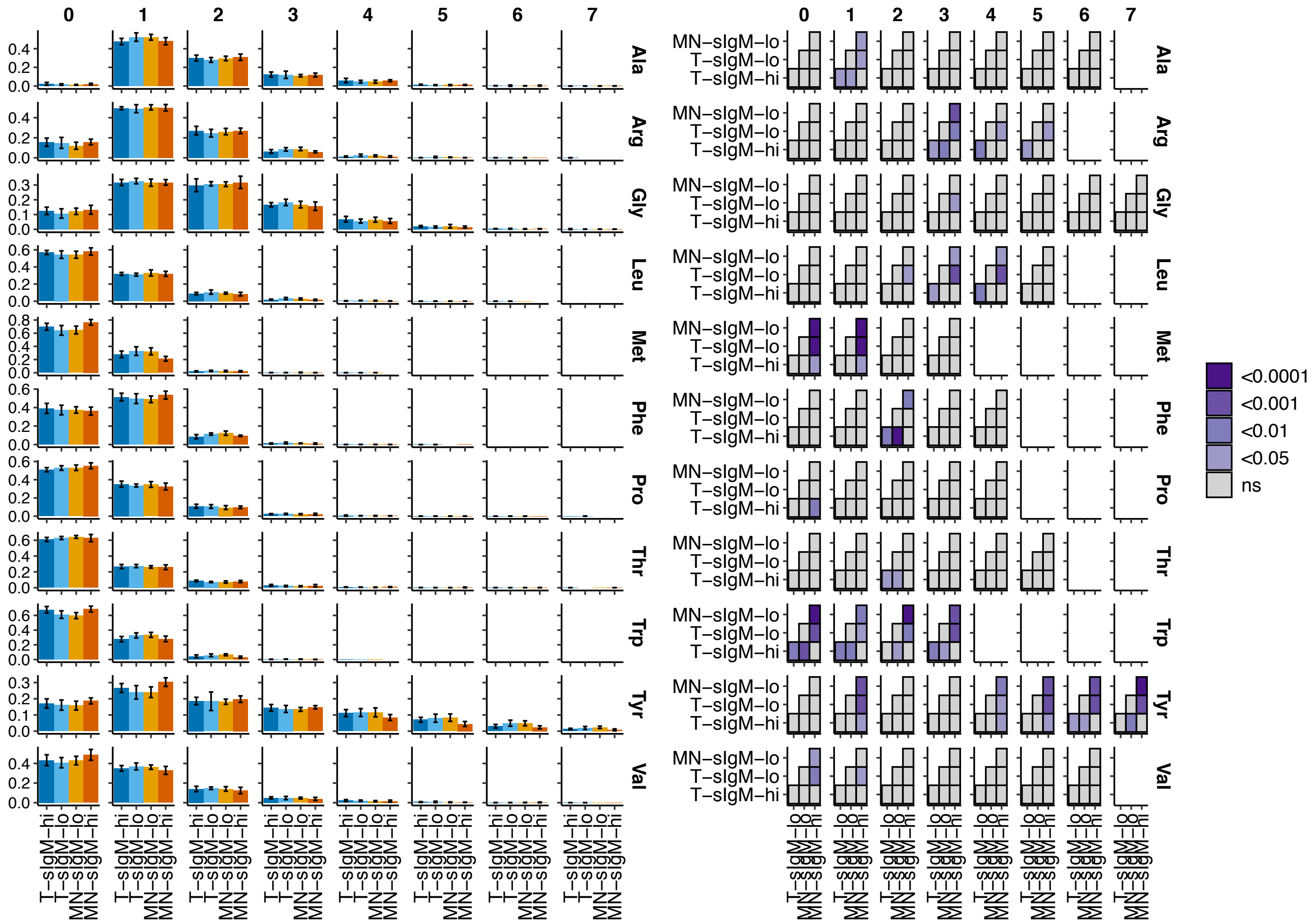

Supplementary Figure 4 Contribution of selective amino acids to CDR3s from the transitional (T) and mature naïve (MN) B cells with high (hi) or low (lo) surface (s) IgM. The upper panel shows the proportion of CDR3s that carry between 0 and 7 residues of the amino acids noted for each row. Bars show mean with error bars showing one standard deviation. The lower panel encodes the statistical significance for the number of residues observed between the different cell subsets. *P*-values for ANOVA Tukey HSD are indicated by the colour scale. Only amino acids that showed a significant difference if the residue count between the four subsets are plotted.

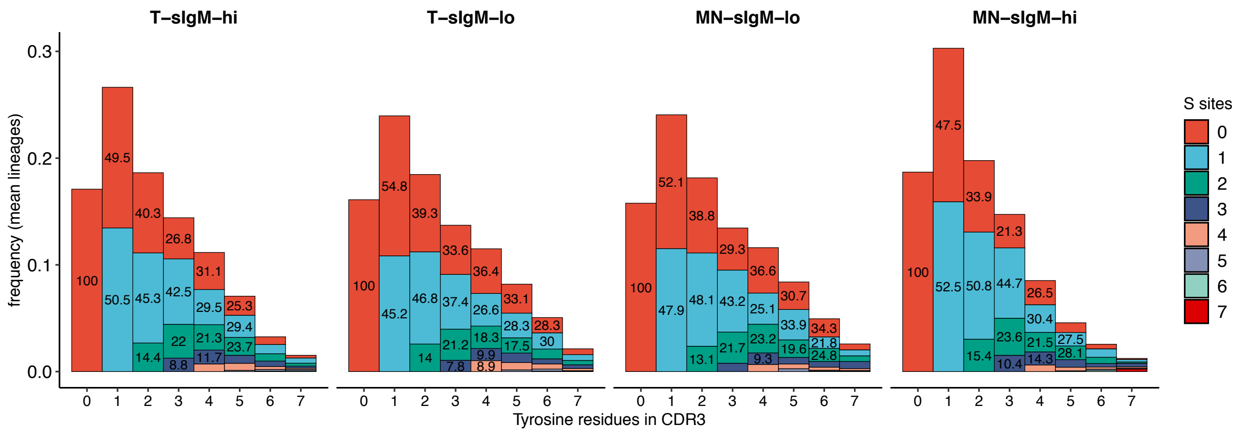

Supplementary Figure 5 Frequency of tyrosine sulfation motifs among CDR3s with varying numbers of tyrosine residues. The number of potential sulfation sites based on sequence motifs were predicted by the GPS-TSP tool for CDR3s with between 0 and 7 tyrosine residues (x-axis). The mean frequency of tyrosine residues within sulfation motifs are indicated by the stacked bars with the colour indicating the number of sites. For example, aqua indicates the proportion of CDR3s with a single tyrosine within a sulfation motif. The mean percentages are printed within the bars. CDR3s from the different subsets do not show difference in sulfation potential of their tyrosine residues.

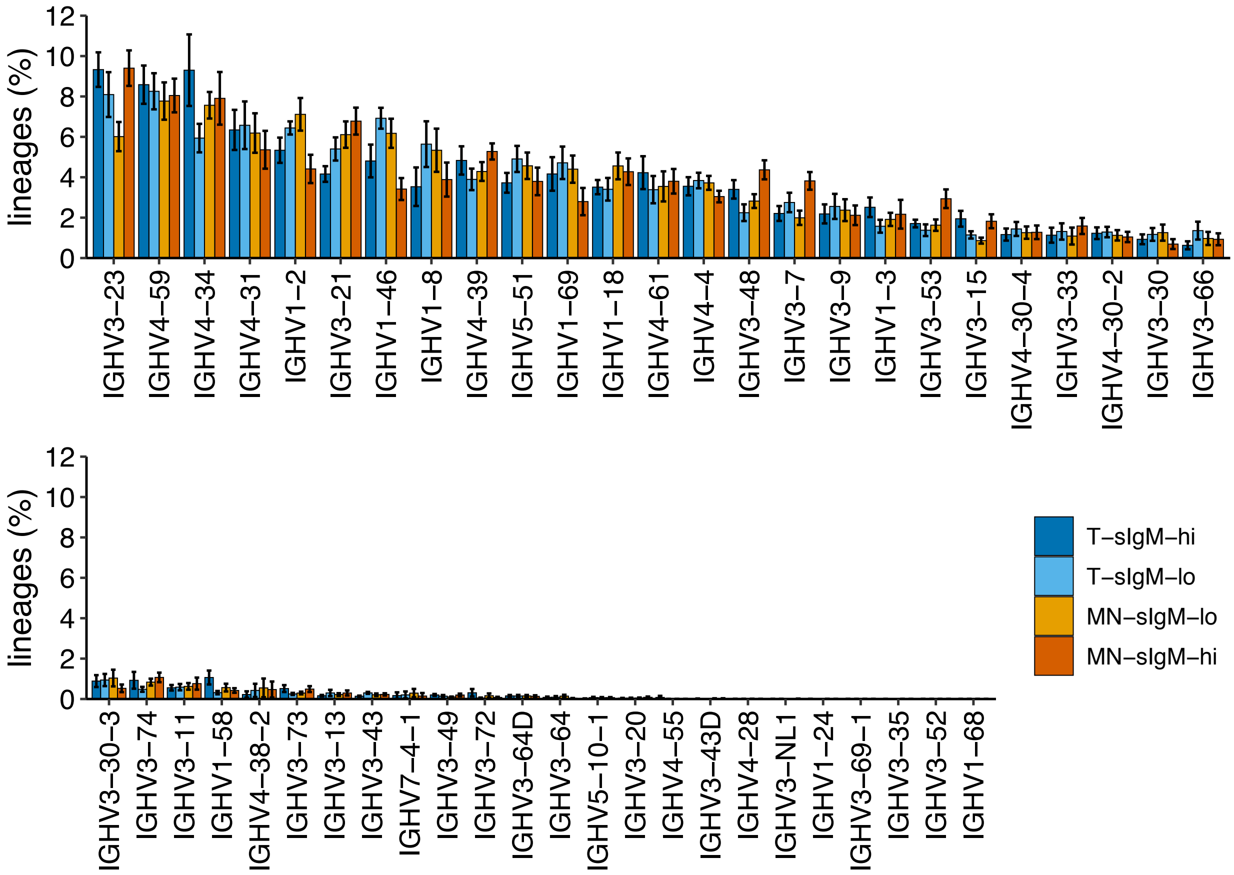

Supplementary Figure 6 IGHV gene usage for transitional (T) and mature naïve (MN) B cells with high (hi) or low (lo) surface (s) IgM. Bars show mean usage across the 12 donors and error bars indicate one standard deviation. IGHV genes are arranged from most to least frequently utilised.

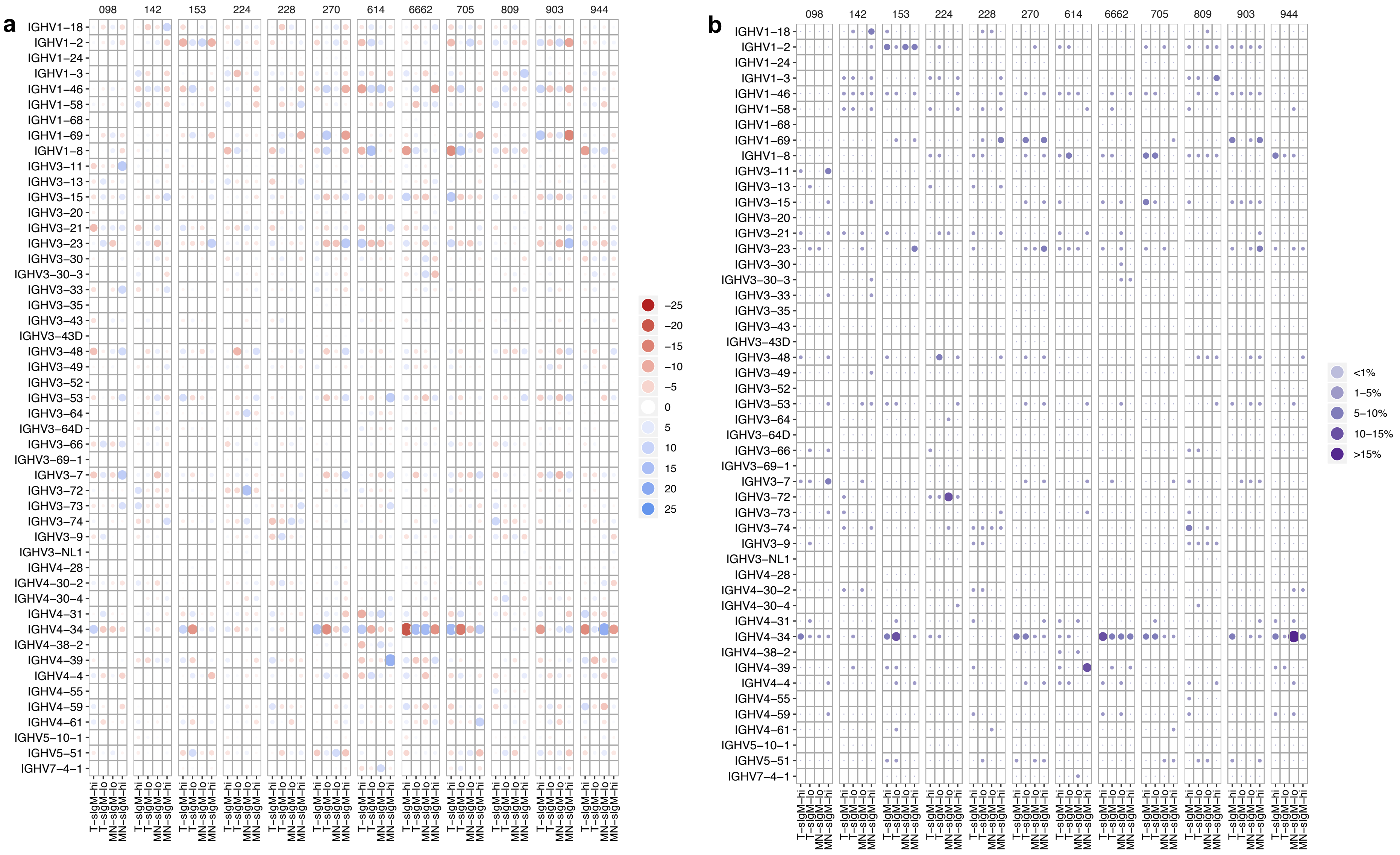

Supplementary Figure 7 Individual donor utilisation of IGHV gene segments usage for transitional and mature naïve B cells with high or low surface IgM. Pearson residuals (**a**) and chi-square statistics (**b**) of the percentage contribution of the residuals to the overall IGHV usage.

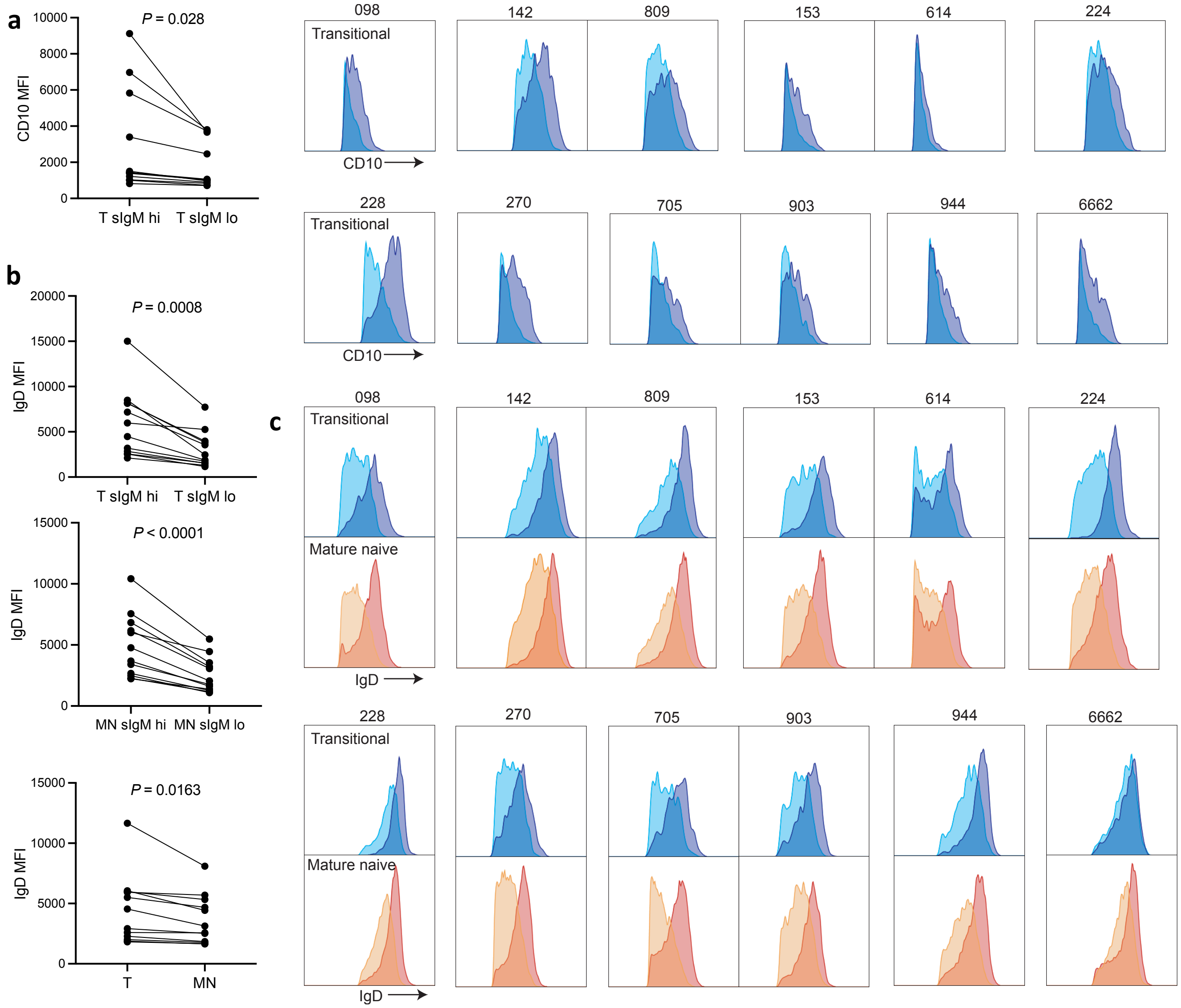

Supplementary Figure 8 Surface CD10 and IgD expression for 12 healthy donors. (**a**) CD10 mean fluorescence intensity (MFI) for IgM^{hi} and IgM^{lo} transitional (T) B cells, each point connected by a solid line represents an individual donor. Histograms of CD10 expression on transitional B cells within upper (dark blue) and lower (light blue) quartiles of surface (s) IgM expression. (**b**) IgD MFI for T and mature naïve (MN) B cells within the upper and lower quartiles of sIgM expression for 12 donors. *P*-values for paired *t*-test comparing MFI for paired data points of different cell populations from each donor. (**c**) Histograms of surface IgD expression for transitional B cells within upper (dark blue) and lower (light blue) quartiles of surface IgM expression and mature naïve B cells within upper (red) and lower (orange) quartiles of surface IgM expression for each donor. Numbers correspond to donor ID in Supplementary Table 1. Histograms are arranged according to experiment, where donors run in the same experiment are grouped.
